# A Superfamily of DNA Transposons Targeting Multicopy Small RNA Genes

**DOI:** 10.1371/journal.pone.0068260

**Published:** 2013-07-09

**Authors:** Kenji K. Kojima, Jerzy Jurka

**Affiliations:** Genetic Information Research Institute, Mountain View, California, United States of America; Keio University, Japan

## Abstract

Target-specific integration of transposable elements for multicopy genes, such as ribosomal RNA and small nuclear RNA (snRNA) genes, is of great interest because of the relatively harmless nature, stable inheritance and possible application for targeted gene delivery of target-specific transposable elements. To date, such strict target specificity has been observed only among non-LTR retrotransposons. We here report a new superfamily of sequence-specific DNA transposons, designated *Dada*. *Dada* encodes a DDE-type transposase that shows a distant similarity to transposases encoded by eukaryotic *MuDR*, *hAT*, *P* and *Kolobok* transposons, as well as the prokaryotic *IS256* insertion element. *Dada* generates 6–7 bp target site duplications upon insertion. One family of *Dada* DNA transposons targets a specific site inside the U6 snRNA genes and are found in various fish species, water flea, oyster and polycheate worm. Other target sequences of the *Dada* transposons are U1 snRNA genes and different tRNA genes. The targets are well conserved in multicopy genes, indicating that copy number and sequence conservation are the primary constraints on the target choice of *Dada* transposons. *Dada* also opens a new frontier for target-specific gene delivery application.

## Introduction

Transposable elements (TEs) are potentially harmful DNA segments capable of reproducing and inserting themselves into genes or other functional genomic regions. Target specificity of TEs for multicopy genes is of great interest because of the stable inheritance and parallel evolution of target-specific TEs as well as their relatively harmless nature [1], [2], [3], [4]. Two non-long terminal repeat (non-LTR) retrotransposons R1 and R2 specifically insert into the 28S ribosomal RNA (rRNA) genes at different sites [1]. Since the rRNA genes are highly repetitive, the deleterious effect of TE insertion disrupting one rRNA gene unit can be negligible although excessive accumulation of insertions could cause developmental defects [5], [6]. R2 has been maintained in the 28S rRNA genes for more than 850 million years, illustrating the success of their survival strategy [2], [3], [7].

To date, such strict target specificity for multicopy genes was observed among non-LTR retrotransposons only [3]. One DNA transposon family, *Pokey*, preferably inserts into the 28S rRNA genes but it also inserts at other genomic locations [8]. Here we report the first target-specific DNA transposon superfamily, designated *Dada*.

Based on sequence similarities between transposases, terminal inverted repeats and target site duplications (TSD), DNA transposons are classified into approximately 20 superfamilies [9]. In the classification applied in Repbase [9], [10], only three superfamilies of DNA transposons (*Helitron*, *Crypton*, and *Zisupton*) lack the DDE-transposases [11], [12], [13]. DDE-transposase represents a very diverse family of protein domains, strictly conserving only three residues, D, D and D/E [9], [10], [14], [15]. DDE-transposase encoded by retroviruses and LTR retrotransposons is called integrase. Some DDE-transposases have been captured to become parts of host systems, and probably the most prominent one is *Transib*-derived recombination activating gene 1 (RAG1), catalyzing V(D)J recombination in vertebrates [16].

*Dada* encodes a protein that is weakly, but significantly similar to DDE-transposases and each family of *Dada* transposons targets specific genes for small nuclear RNA (snRNA) or transfer RNA (tRNA). The similarity between targets of *Dada* and target-specific non-LTR retrotransposons implies universal constraints in the target specificity of TEs. Due to its target specificity, *Dada* can potentially be used for gene delivery.

## Results

### Dada, a New Superfamily of DNA Transposons Encoding DDE Transposases

In our systematic survey for repetitive sequences from available genome sequences, we found two related repetitive sequences from *Danio rerio* and *Daphnia pulex*. Using these nucleotide sequences and their encoding protein sequences as queries, we performed blast searches against eukaryotic genomic and EST databases, and found related sequences in diverse eukaryotes including animals, fungi, plants and monocellular eukaryotes (Table 1). Several, nearly identical copies of these sequences were present in a single genome. We generated consensus sequences when more than three copies with over 90% identity are available. If there were less than three copies, the single copy or the copy with the longest open reading frame was used for further analysis. The proteins encoded by these repetitive sequences show a weak but significant similarity to DDE-transposases (below in this section). Finally, they are often inserted into specific types of RNA genes with TSD (the next section and thereafter). From these observations, we concluded that they represent a new group of TEs, and named these TEs as “*Dada*” or “*Dada* transposons” from *Danio* and *Daphnia*, the genus names of organisms in which they were found originally, and their transposases are referred to as “Dada transposases.”

**Table 1:** *Dada* transposons found in this study.

Name	Organism	Consensus	Representative sequence
*Dada-U6_DR*	*Danio rerio*	Yes	NW_001878847 57919-66821
*Dada-U6N1_DR*	*Danio rerio*	Yes	NW_003040715 16813-19219
*Dada-U6_SS*	*Salmo salar*	No	AGKD01002144 12916-4875
*Dada-U6_GA*	*Gasterosteus aculeatus*	Yes	AANH01010141 100155-107670
*Dada-U6_OL*	*Oryzias latipes*	Yes	NW_004091833 8077-316
*Dada-U6_DPu*	*Daphnia pulex*	Yes	ACJG01005766 2506-1
*Dada-U6_CT*	*Capitella teleta*	Yes	AMQN01000286 22970-20257
*Dada-U6_CGi*	*Crassostrea gigas*	No	AFTI01007226 21538-15486
*Dada-U1A_DR*	*Danio rerio*	Yes	NC_007115 46815275-46826264
*Dada-U1B_DR*	*Danio rerio*	Yes	NC_007115 42796214-42805757
*Dada-tA_DR*	*Danio rerio*	Yes	NC_007136 25985664-25976995
*Dada-tA_OL*	*Oryzias latipes*	Yes	NW_004091117 7850-4929
*Dada-tL_DR*	*Danio rerio*	Yes	NW_003336270 130291-119937
*Dada-1_TN*	*Tetraodon nigroviridis*	Yes	CAAE01008492 86683-81904
*Dada-1_FR*	*Fugu rubripes*	Yes	NW_004071127 553-3103
*Dada-1_DL*	*Dicentrarchus labrax*	Yes	CABK01011283 1434-95
*Dada-1_GM*	*Gadus morhua*	Yes	CAEA01545225 2-3072
*Dada-1_ON*	*Oreochromis niloticus*	Yes	NT_167802 200659-197454
*Dada-1_BF*	*Branchiostoma floridae*	Yes	NW_003101470 208198-217431
*Dada-1_CSa*	*Ciona savignyi*	Yes	AACT01042470 4966-11081
*Dada-1_CI*	*Ciona intestinalis*	Yes	NW_004190570 12920-6453
*Dada-1_CGi*	*Crassostrea gigas*	No	AFTI01018005 30202-24790
*Dada-1_NV*	*Nematostella vectensis*	Yes	NW_001833510 41468-38072
*Dada-1_MB*	*Monosiga brevicollis*	Yes	NW_001865079 246704-249422
*Dada-1_LB*	*Laccaria bicolor*	Yes	NW_001889872 3424403-3432175
*Dada-2_LB*	*Laccaria bicolor*	Yes	NW_001889876 1244316-1249967
*Dada-1_ES*	*Ectocarpus siliculosus*	Yes	CABU01001069 5888-1201
*Dada-1_CV*	*Chlorella variabilis*	Yes	ADIC01000572 92694-99664
*Dada-tL_PMar*	*Perkinsus marinus*	Yes	NW_003212056 26485-32261
*Dada-tIA_PMar*	*Perkinsus marinus*	No	NW_003214659 491397-486732
*Dada-tIB_PMar*	*Perkinsus marinus*	No	NW_003209212 4075-9263
*Dada-tG_PMar*	*Perkinsus marinus*	No	NW_003210318 27228-41234
*Dada-tY_PMar*	*Perkinsus marinus*	No	NW_003214682 62629-66909
*Dada-2_PMar*	*Perkinsus marinus*	Yes	NW_003210480 132081-135066
*Dada-3_PMar*	*Perkinsus marinus*	No	NW_003216097 8555-4510
*Dada-4_PMar*	*Perkinsus marinus*	No	NW_003209437 33853-30539

All sequences are deposited in Repbase Update (http://www.girinst.org/repbase).

**Figure 1:**
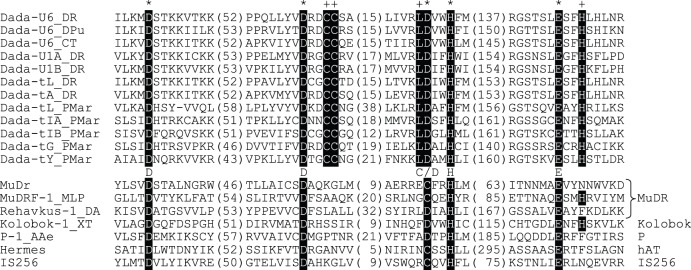
DDE-transposase motifs of Dada transposases aligned with those of other transposases. The catalytic DDE triad and C/DxxH motif are indicated by asterisks while other residues conserved among all *Dada* families are marked by plus symbols. Numbers in parentheses indicate the lengths of sequences between motifs.

While blast search using Dada transposases as queries did not match any transposases, the secondary structure-based homology search program HHpred (http://toolkit.tuebingen.mpg.de/hhpred/) detected a weak similarity of Dada transposases to retroviral integrases (avian sarcoma virus and human immunodeficiency virus type 1), and to the bacteriophage Mu transposase (data not shown). We identified the conserved catalytic triad (DDE) and a DxxH motif following the second conserved D based on the alignment with other transposases (Fig. 1). The DxxH (or CxxH) motif is present in transposases from four eukaryotic DNA transposon superfamilies (*hAT*, *Kolobok*, *P* and *MuDR*), and from the bacterial *IS256* transposons [10], [15]. *Dada* transposons belong to a new superfamily of DNA transposons.

The length of complete *Dada* transposons ranges from 4666 to 10979 bp. As an instance, *Dada-U6_DR* is 8963 bp in length. Programs predicting exon-intron boundaries suggested that *Dada-U6_DR* contains 11 exons encoding a protein whose length is 1402 amino acids. All *Dada* transposons except those from *Perkinsus marinus* contain introns. The three catalytic residues of DDE-transposase are D567, D635 and E811 in the *Dada-U6_DR* transposase. All Dada transposases contain N-terminal CCCC zinc finger motif, which corresponds to C389, C394, C429 and C432 in the *Dada-U6_DR* transposase, and a C-terminal CCHC zinc finger motif, which corresponds to C1359, C1362, H1372 and C1381 in the *Dada-U6_DR* transposase. Protein alignment of Dada transposases is available as Dataset S1.

*Dada* transposons from *Laccaria bicolor* and *Ectocarpus siliculosus* encode a DEDDy-type DnaQ-like 3′–5′ exonuclease domain (Fig. S1). It is located between the second catalytic D and the DxxH motif and conserved all four catalytic residues (DEDD). These exonucleases likely process the cleaved 3′ ends exposed during transposition.

### Dada-U6 Transposons Targeting U6 snRNA Genes

**Figure 2:**
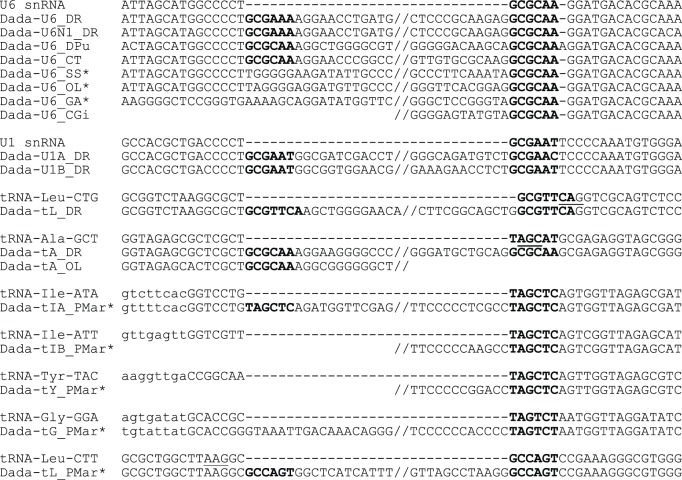
Insertion sites of *Dada* families. Flanking sequences including TSD and terminal sequences of *Dada* transposons are aligned with target RNA genes. TSD sequences are in boldface. Asterisk indicates that the 5′ terminus was determined based on one copy. Anticodon is underlined. Lower cases represent non-genic sequence.

All *Dada* transposons with clearly definable termini were inserted into specific types of small RNA genes with short TSD (Fig. 2). Their target genes and the host species are reflected in the nomenclature of different *Dada* families. For example, *Dada-U6_DR* from zebrafish *Danio rerio* is located between two U6 fragments corresponding to the gene sequence coordinates 1–70 and 65–104 implying ^65^GCGAAA^70^ or ^65^GCGCAA^70^ as TSD. The transposase is encoded in the opposite direction relative to the orientation of the U6 snRNA genes. Internally deleted derivatives of *Dada-U6_DR*, named *Dada-U6N1_DR,* are also inserted at the same site. They share the 5′ 231 bps and the 3′ 1567 bps with *Dada-U6_DR*.

We also found *Dada* transposons inside the U6 arrays from salmon (*Salmo salar*), medaka (*Oryzias latipes*), stickleback (*Gasterosteus aculeatus*), water flea (*Daphnia pulex*), oyster (*Crassostrea gigas*) and a polycheate worm (*Capitella teleta*; Fig. 2). *Dada-U6* elements from three distantly related species (zebrafish, water flea and *Capitella*) were characterized in depth. They are mostly inserted into U6 snRNA genes with 6-bp TSD (GCGCAA or GCGAAA; Fig. S2). Several of them are flanked by non-U6 sequences but never at both ends. Notably, the Dada transposases inside the U6 genes from *Capitella* are encoded in the same orientation as U6 genes.

**Figure 3:**
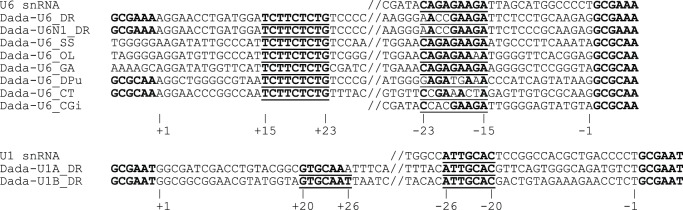
Sub-terminal inverted repeats of *Dada-U6* and *Dada-U1*. Both terminal sequences of *Dada* transposons with TSD are shown. U6 and U1 snRNA genes are also aligned. TSD are in boldface type and sub-terminal inverted repeats are in boldface and underlined.

Based on the comparison of *Dada*-inserted and uninserted U6 genes, we easily recognized the termini of *Dada* transposons. However, we did not find any terminal inverted repeats in the *Dada* transposons. Instead we identified 9-bp sub-terminal inverted repeats (TCTTCTCTG and CAGAGAAGA) shared among all *Dada-U6* families (Fig. 3). Moreover, we found the sequence CAGAGAAGA in the U6 snRNA genes. They are all at the same distance from the TSD and we speculate that these short inverted repeats may be involved in target site recognition.

### Dada-U1 Transposons Targeting U1 snRNA Genes

*Dada* transposons are also present in U1 snRNA genes. Two families of *Dada* transposons (*Dada-U1A_DR* and *Dada-U1B_DR*) from *Danio rerio* are inserted in U1 snRNA genes in the same direction at identical sites. They appear to be flanked by the 8-bp TSD (CTGCGAAT or CTGCGAAC; Fig. 2). However, the actual TSD is likely to be GCGAAT/GCGAAC for the following reasons. First, tandemly inserted *Dada-U1A_DR* and *Dada-U1B_DR* copies on chromosome 12 are separated by GCGAAT (Fig. S3). Second, two *Dada-U1A_DR* copies on chromosome 3 are arrayed in tandem without any additional nucleotides between them, assuming GCGAAT/GCGAAC as TSD (Fig. S3). Finally, *Dada-U6* transposons are flanked by GCGAAA or GCGCAA TSD following the 5′ flanking CT (Fig. 2). In the case of *Dada-U1* transposons, the sequence GCGAAT/GCGAAC follows the 5′ flanking CT. While we cannot rule out the possibility of 8-bp TSD, we propose a 6-bp GCGAAT/GCGAAC as the TSD of *Dada-U1A_DR* and *Dada-U1B_DR*. Like *Dada-U6* transposons, *Dada-U1* transposons do not have terminal inverted repeats but have short sub-terminal inverted repeats (GTGCAAT and ATTGCAC) shared between the *Dada-U1* transposons (Fig. 3). We also found the sequence ATTGCAC in the U1 snRNA genes at the same distance from the TSD sites.

### Dada-tL_DR Transposons Targeting tRNA-Leu Genes

**Figure 4:**
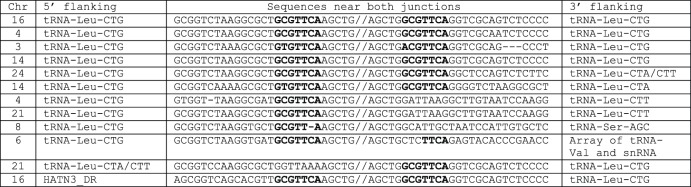
Flanking sequences of *Dada-tL_DR* insertions. Chromosome numbers, the annotations of 5′ and 3′ flanking sequences, and the sequences near the 5′ and 3′ junctions of 12 *Dada-tL_DR* insertions are shown. TSD are shown in boldface.

*Dada* transposons also target tRNA genes from zebrafish. One *Dada* family (*Dada-tL_DR*) is located inside of tRNA-Leu genes while the other (*Dada-tA_DR*) is present inside of tRNA-Ala genes. In the sequenced genome of zebrafish, there are 12 copies of *Dada-tL_DR* with both termini, some of which have internal deletions and/or insertions (Fig. 4). Four of them are inserted in tRNA-Leu-CTG with GCGTTCA TSD, or their variants (see rows 1–4 in Fig. 4). The 5′ and 3′ flanking sequences of the remaining insertions did not come from the same gene. One end of each inserted element is always flanked by tRNA-Leu-CTG, whereas the other end is flanked by tRNA-Leu-CTA, tRNA-Leu-CTT, or tRNA-Ser-AGC gene. It has also been found to be flanked by spacer of the array of tRNA-Val and snRNA genes, or a sequence inside the *HATN3_DR* transposon (see the rows 5–12 in Fig. 4). The GCGTTCA sequence is always present at the side of tRNA-Leu-CTG, but sometimes absent from the other side.

Assuming that the original *Dada-tL_DR* was specifically inserted into a tRNA-Leu-CTG with GCGTTCA TSD, we propose a possible mechanism underlying these insertions. If, for example, only one end of the *Dada-tL_DR* is cleaved and rejoined to a fragment of tRNA-Ser-AGC, probably catalyzed by the Dada transposase, but the other end is not, this copy becomes sandwiched between a fragment of tRNA-Leu-CTG and a fragment of tRNA-Ser-AGC. This mechanism is basically identical to the “one-ended transposition” reported in V(D)J recombination [17]. Similar mechanism can also be applied to *Dada-U6* transposons flanking non-U6 sequences (Fig. S2).

The targeted tRNA genes are present in high copy numbers. There are 280 intact copies of zebrafish tRNA-Leu-CTG and 398 intact copies of tRNA-Leu-CTT or tRNA-Leu-CTA that are >95% identical to their respective consensus sequences over >95% of their length. Similarly, there are 363 intact copies of tRNA-Ser-AGC in the zebrafish genome. These numbers are similar to the numbers of tRNA genes reported in Genomic tRNA database (http://gtrnadb.ucsc.edu/).

### Dada-tA Transposons Targeting tRNA-Ala Genes

*Dada-tA_DR* insertions were found in tRNA-Ala-GCT genes, but the *Dada-tA_DR* insertions are flanked by GCGCAA TSD, instead of TAGCAT in the five out of the six full-length copies found (Figs. 2 and S4). The medaka *O. latipes* also contains *Dada-tA* copies (*Dada-tA_OL*) adjacent to GCGCAA. We confirmed that there is no intact tRNA gene containing GCGCAA at the corresponding site in either zebrafish or medaka. The data suggest that *Dada-tA* replaced TAGCAT with GCGCAA upon integration by an unknown mechanism. The GCGCAA sequences might have been the ancestral TSD of *Dada-tA_DR* because their relatives are flanked by either GCGCAA/GCGAAA (*Dada-U6*) or GCGAAT (*Dada-U1*). There are 80 copies of tRNA-Ala-GCT in the zebrafish genome (Genomic tRNA database).

### Dada Transposons Targeting tRNA Genes from Perkinsus Marinus

*Dada* transposons targeting tRNA genes were also found in the oyster parasite *Perkinsus marinus* (Table 1). These insertions are present in different tRNA genes: tRNA-Ile, tRNA-Leu, tRNA-Gly and tRNA-Tyr, but each family of *Dada* transposons targets only its family-specific tRNA genes (Fig. 2). Likewise in the case of *Dada-U1A_DR* and *Dada-U1B_DR*, we propose that the TSD of *Dada-tIA_PMar* are TAGCTC instead of TAGCTCAG. Putative TSD of *Dada-tIA_PMar*, *Dada-tIB_PMar* and *Dada-tY_PMar* represents identical TAGCTC sequence, which is a part of the A box of the polymerase III promoter.

We counted the tRNA genes with sequences >95% identical to their consensus sequences and with length >95% of their consensus sequences in the genome shotgun scaffold set (AAXJ01.fasta, http://0-www.ncbi.nlm.nih.gob.ilsprod.lib.neu.edu/Traces/wgs). We found 9 tRNA-Ile-ATA, 46 tRNA-Ile-ATT, 116 tRNA-Gly-GGA, 23 tRNA-Tyr-TAC and 349 tRNA-Leu-CTT genes. The actual tRNA copy numbers per haploid genome may be smaller than the numbers above since we found 1–3 sequences (1.5 on average) corresponding to a single-copy gene in the scaffold set (data not shown).

### Recent Activity of Dada Transposons

We found three full-length copies for each family of *Dada-U6_DR*, *Dada-U1A_DR* and *Dada-U1B_DR*. They are >99% identical to one another and encode a long protein including a DDE-transposase domain, which indicates their recent transposition activity. Without recent transposition, passive duplication along with their targets could not maintain the protein coding capacity. One EST sequence, CT606019 from zebrafish, corresponds to the protein-coding sequence of *Dada-U6_DR*. EST sequences from *Pimephales promelas* (fathead minnow), medaka and *Ciona intestinalis* support the expression of proteins encoded by *Dada* transposons.

## Discussion

### Target Specificity of DNA Transposons

Target sequence-specific integration of TEs is observed almost exclusively in non-LTR retrotransposons. Many retrotransposons show specific integration of certain types of repetitive sequences including telomeric repeats, microsatellites and multicopy RNA genes [3], [4]. In the previous article [3], it was proposed that genes for rRNA, tRNA and snRNA are ideal targets for target-specific TEs because of their high copy numbers and sequence conservation. The characterization of *Dada* transposons in a variety of snRNA and tRNA genes is consistent with this assumption. The similarity of targets for target-specific non-LTR retrotransposons and *Dada* indicates that a highly similar selective pressure selects the targets for both non-LTR retrotransposons and DNA transposons.

Aside from the target sequence specificity observed among the non-LTR retrotransposons described above, which recognize target DNA sequences directly, there is another type of target specificity, which is mediated by interactions between TE proteins and the host DNA-binding proteins. This type of target specificity is observed in *TRE5-A* non-LTR retrotransposons from *Dictyostelium discoideum* and *Tf1* LTR retrotransposons from *Schizosaccharomyces pombe* [18], [19]. Although these retrotransposons target specific types of sequences such as tRNA genes or RNA polymerase II promoters, they are not inserted at specific positions inside of their targets, but at a distance close to the targets. *Dada* transposons are inserted at specific sites inside their target sequences, which resemble target-specific non-LTR retrotransposons directly recognizing the DNA sequences.

Zebrafish is the species with many *Dada* transposons and large numbers of tRNA and snRNA genes. Zebrafish carries 12794 tRNA genes, almost 25 times as many as humans (513 tRNA genes; Genomic tRNA database, http://gtrnadb.ucsc.edu/). The copy numbers of intact U6 and U1 snRNA genes in zebrafish are 654 and 297, respectively (>95% identity to the consensus, and >95% of length). They far exceed the corresponding numbers in the human genome, which are 44 and 16 [20]. The huge numbers of RNA genes in the zebrafish genome enable *Dada* transposons to be maintained with little impact. Therefore, it is of little surprise that the zebrafish genome maintains many target-specific TEs in addition to *Dada* transposons: *R2* for 28S rRNA genes, *Mutsu* for 5S rRNA genes, *Keno* for U2 snRNA genes, and *Dewa* for the spacer of tRNA-Leu [3].

*Perkinsus marinus* harbors five families of *Dada* transposons, all specifically inserted into tRNA genes. Although the numbers of tRNA genes, especially tRNA-Ile and tRNA-Tyr, are much smaller than those of zebrafish, they are quite large among parasitic monocellular eukaryotes. We found more than 500 copies in five types of tRNA genes from *P. marinus*, which exceeds the numbers of total tRNA genes of other parasitic eukaryotes, which are generally below 100 [21]. It is likely that insertions of *Dada* transposons into parts of tRNA genes hardly affect the fitness of *P. marinus*.

### Recognition of Target Sequences by Dada Transposases

**Figure 5:**
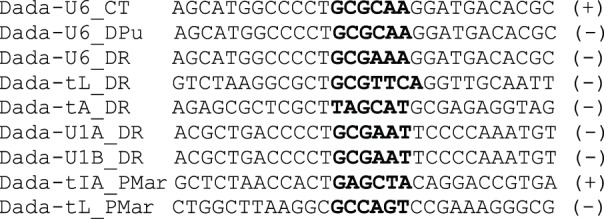
Alignment of insertion sites and TSD of *Dada* families. TSD are shown in boldface. Plus symbol indicates that the coding direction of Dada transposase is the same as of the RNA genes while minus symbol indicates the opposite.

A general feature associated with TE insertions is generation of flanking TSD. The size and sequence of TSD are the diagnostic characters of each DNA transposon superfamily, which reflect the mechanism of transposition. The length of *Dada* TSD is consistent with the similarity of *Dada* to *hAT*, *Kolobok*, *P* and *MuDR* (Fig. 5). These groups of DNA transposons generate long TSD between 4 to 10 bp [9]. The length of TSD of *Dada* (6–7 bp) falls into this range.

Generating longer TSD appears to be linked to recognition of longer target sequences. Transposons belonging to the *P* and *hAT* superfamilies, which generate ∼8-bp TSD, tend to be integrated into a 14-bp sequence motif that includes TSD inside, while *Mariner/Tc1* transposons, which generate 2-bp TSD, recognize sequences up to 8 bp [22], [23], [24]. Given the similarity of Dada transposases to transposases of the *P* and *hAT* superfamilies, Dada transposases would recognize longer sequence motifs. It is essential to target certain RNA genes in the genome because longer sequence motif is less likely to be present outside of target repetitive sequences by chance.

There is a clear sequence similarity among target sequences of *Dada* transposons (Fig. 5). Four out of five insertion sites from zebrafish share CTGCG in which GCG is a part of TSD. Targets of *Dada-U6_DR* and *Dada-U1A_DR*/*Dada-U1B_DR* share a longer sequence motif CCCCTGCGAA in which GCGAA is a part of TSD. Furthermore, we could see a similarity even between targets of *Dada-tIA_PMar* and animal *Dada* families despite the diversity of their host species and the difference of target RNA genes. Overall, the sequences at one side (corresponding to the upstream sequences in Fig. 5) are more conserved among different families than those of the other side, indicating that the cleavage of one strand by Dada transposases is more strictly defined than the other.

### Potential Usage of Transgenic Vectors of Dada Transposons

Due to their target specificity, *Dada* transposons can be used as vectors for transgenesis. Transgenesis systems have been established for *Sleeping Beauty*, *piggyBac* and *Tol2*, but their nearly random integration is a threat to gene therapy, having a potential to disrupt genes or interfere with gene expression [25]. Several methods to integrate DNA into a specific locus are being developed. One is a combination of DNA transposons and a targeting domain originated from DNA-binding proteins such as zinc finger motifs [26]. Another is the usage of target-specific non-LTR retrotransposons like R1 and SART1 [27]. The identification of *Dada* opens a new opportunity for development of a safer therapeutic vector.

## Methods

### Data Sources

Genomic sequences of various species were obtained mostly from GenBank, and sequences of known TEs were obtained from Repbase [10] (http://www.girinst.org/repbase).

### Sequence Analysis

*Dada-U6_DR* and *Dada-U6_DPu* were detected by systematic screening of new repetitive sequences using custom-made scripts based on the methods described before [28]. Characterization of new *Dada* transposons was achieved by repeated BLAST [29] and CENSOR [30] searches using genomic sequences of various species with *Dada* transposons as queries. All analyses were done with default settings. The consensus sequences of the *Dada* transposons were derived using the majority rule applied to the corresponding sets of aligned copies. Exon-intron boundaries were predicted with the aid of SoftBerry FGENESH: (http://linux1.softberry.com/berry.phtml?topic=fgenesh&group=programs&subgroup=gfind) and GENEID (http://genome.crg.es/geneid.html). The sequence alignments of the predicted protein-coding sequences with available EST sequences and with the predicted protein sequences of different families of *Dada* transposons were done to improve the prediction. We used MAFFT [31] with the linsi option to align protein sequences of various *Dada* transposons. The sequences of TEs reported in this work are deposited in Repbase Update [10] (http://www.girinst.org/repbase).

## Supporting Information

Figure S1 Alignment of exonuclease domains of *Dada* transposons with other DEDDy-type exonucleases. Conserved residues DEDDy are in red. Accession numbers are as follows. WRN-Exo_HS, 2FC0_A; MUT-7_CE, CAA80137; RRP6_HS, AAH73788; RNASED_EC, ACI82335; Klenow_EC, 1QSL_A; and T7DNAPol, 1×9S_A.
(PDF)

Figure S2 Insertion sites of *Dada-U6* transposons. TSD are colored in red and *Dada* transposons are in blue.
(PDF)

Figure S3 Tandem insertions of *Dada-U1A_DR* and *Dada-U1B_DR* transposons. The sequences of *Dada-U1A_DR* are colored in blue, of *Dada-U1B_DR* in magenta, and of TSD in red.
(PDF)

Figure S4 Insertions of *Dada-tA_DR* and *Dada-tA_OL*. TSD are colored in red and *Dada* transposons are in blue. Anticodons in the tRNA genes are underlined.
(PDF)

Dataset S1 Full-length protein alignment of Dada transposases in fasta format.
(FA) Click here for additional data file.
