# Supplementary material for: A Superfamily of DNA Transposons Targeting Multicopy Small RNA Genes
Source: PLoS One. 2013 Jul 9;8(7):e68260. doi: 10.1371/journal.pone.0068260 (PMC3706591; doi:10.1371/journal.pone.0068260)
Supplement: Figure S1 — Alignment of exonuclease domains of Dada transposons with other DEDDy-type exonucleases. Conserved residues DEDDy are in red. Accession numbers are as follows. WRN-Exo_HS, 2FC0_A; MUT-7_CE, CAA80137; RRP6_HS, AAH73788; RNASED_EC, ACI82335; Klenow_EC, 1QSL_A; and T7DNAPol, 1×9S_A. (PDF) [file pone.0068260.s001.pdf]

**Figure S1.**

```
Dada-1_LB    LVVGLDTEWDVDLSA----------RRQGVPDHRKTAIMQIAYGNE-VWIFQLSE----H
Dada-2_LB    VVIGFDTEWNVD-------------RTPGNSRQGKTAVVAISFESH-IFVLQISQ----Y
Dada-1_ES    NVLGLDCEWEPS-------------LAGTTP--NPVSTVQLSLPDGTAYCFQLQRGNK-K
WRN-EXO_HS   DVVGFDMEWPPL-------------YNRGKL--GKVALIQLCVSESKCYLFH-------V
MUT-7_CE     VYVGFDSEWKPS--------------NLTAVHDSKIAIIQLFFKNC-VWLVDCVELEKAN
RRP6_HS      QEFAVNLE-----------------HHSYRSFLGLTCLMQISTRTE-DFIID-------T
RNASED_EC    PAIALDTE-----------------FVRTRTYYPQLGLIQLFDGEH-LALID-------P
Klenow_EC    PVFAFDTETDSLDNISANLVGLSFAIEPGVAAYIPVAHDYLDAPDQ--------------
T7DNAPol     MIVS-DIEANALLESVTK-------FHCGVIYDYSTAEYVSYRPSD--------------

Dada-1_LB    IANGSFPAQLSTFLANSQILKVGRNVLLDLKN------LQEDSESSTPFVGGIDLGRLAK
Dada-2_LB    TKAGKLPVAIKNILADERILKVGRGIKNDLKR------LQTEGNIKDPFPSFVELAPLAK
Dada-1_ES    TTSSNFPKALQNLLENPSIAKVGVNINSDATY------LERDYGIE--VANTVDLRTYAR
WRN-EXO_HS   SSMSVFPQGLKMLLENKAVKKAGVGIEGDQWK------LLRDFDIK--LKNFVELTDVAN
MUT-7_CE     MADDWWQKFASRLFGDSPVKVVGFDMRNDLDAMATIPALKSSMKIED-TKNAFDLKRLAE
RRP6_HS      LELRSDMYILNESLTDPAIVKVFHGADSDIEW------LQKDFGLY--VVNMFDTHQAAR
RNASED_EC    LGI-TDWSPLKAILRDPSITKFLHAGSEDLEV------FLNVFGEL--PQPLIDTQILAA
Klenow_EC    ISRERALELLKPLLEDEKALKVGQNLKYDRG-------ILANYGIE--------LRGIAF
T7DNAPol     --FGAYLDALEAEVARGGLIVFHNGHKYDVPALTKLAKLQLNREFHLPRENCIDTLVLSR

Dada-1_LB    EK-------NVVSDARASLGDLCIKTLSCILP-------------KDLNIRI--------
Dada-2_LB    KC-------HIAPNARVSLTELSALVLGFKLN-------------KDDAIRV--------
Dada-1_ES    QC--------WVETPSRSLAGMASSLLGRQLP-------------KDPVIRS--------
WRN-EXO_HS   KK--------LKCTETWSLNSLVKHLLGKQLL-------------KDKSIRC--------
MUT-7_CE     NVCDIDMEILELPKKTFKLADLTHYLLGLELD-------------KTE--QC--------
RRP6_HS      LL----------NLGRHSLDHLLKLYCNVD---------------SNKQYQL--------
RNASED_EC    FC---------GRPMSWGFASMVEEYSGVTLD-------------KSE--SR--------
Klenow_EC    DT-------M-----------LESYILNSVAG-------------RHDMDSL--------
T7DNAPol     LI--------HSNLKDTDMGLLRSGKLPGALEAWGYRLGEMKGEYKDDFKRMLEEQGEEY

Dada-1_LB    --SPDW--------------SG-PLTDEQI------QYAALDAWASLKIYEKL
Dada-2_LB    --STEW--------------ENINLSQDQI------MYIAQDAHASKCIYEKL
Dada-1_ES    --S-RW--------------SS-PLSDNQE------VAKFQDPIFS-------
WRN-EXO_HS   --S-NW--------------SKFPLTEDQK------LYAATDAYAGFIIYR--
MUT-7_CE     --S-NW--------------QCRPLRKKQI------VYAALDAVVVVETFKKI
RRP6_HS      --A-DW--------------RIRPLPEEML------SYARDDTHYLLYIYDKM
RNASED_EC    --T-DW--------------LARPLTERQC------EYAAADVWYLLPITAKL
Klenow_EC    --AERWLKHKTITFEEIAGKGKNQLTFNQIALEEAGRYAAEDADVTLQLHLKM
T7DNAPol     VDGMEW----------------WNFNEEMM------DYNVQDVVVTKALLEKL
```
