# Supplementary material for: A Superfamily of DNA Transposons Targeting Multicopy Small RNA Genes
Source: PLoS One. 2013 Jul 9;8(7):e68260. doi: 10.1371/journal.pone.0068260 (PMC3706591; doi:10.1371/journal.pone.0068260)
Supplement: Figure S2 — Insertion sites of Dada-U6 transposons. TSD are colored in red and Dada transposons are in blue. (PDF) [file pone.0068260.s002.pdf]

Figure S2.

Dada-U6_DR(Danio rerio)

```
U6 snRNA                AGAAGATTAGCATGGCCCCT------------------------------------------------GCGAAAGGATGACACGCAAATCCGTG
chr3                    AGAAGATTAGCATGGCCCCTGCGAAAAGGAACCTGATGGATCTTCT//CGAAGATTCTCCCGCAAGAGGCGCAAGGATGACACGCAAATCCGTG
chr3                    ATGTGAAATCCCTGTTCCCTGCGAAAAGGAACCTGATGGATCTTCT//CGAAGATTCTCCTGCAAGAGGCGCAAGGATGACACGCAAATCCGTG
Zv7_scaffold2498        AGAATATTAGCATGGCCCCTGCGAAAAGGAACCTGATGGATCTTCT//CGAAGATTCTCCTGCAAGAGGCGAAAGGATGACACGCAAATCCGTG
chr4                    AGAAGATTAGCATGGCCCCTGCGAAAAGGAACCTGATGGATCTTCT//
Zv7_scaffold2553        AGAATATTAGCATGGCCCCTGTGAAAAGGAACCTGATGGATCTTTT//
Zv7_scaffold2558        AGAAGATTAGCATGGCCCCTGCGCAAAGGAACCTGATGGATCTTCT//
Zv7_NA393                                                             //CGAAGATTCTCCCGCAAGAGGCGAAAGGATGACACGCAAATCCGTG
chr4                                                                  //CGAAGATTCTCCAGCAAGAGGCGAAAGGATGACACGCAAATCCGTG
```

Dada-U6N1_DR(Danio rerio)

```
U6 snRNA                AGAAGATTAGCATGGCCCCT------------------------------------------------GCGAAAGGATGACACGCAAATCCGTG
Zv7_scaffold2602        AGAAGATTAGCATAGCCCCTGGGAAAAGGAACCTGATGGATCTTCT//CGAAGATTCTCCCGCAAGAGGCGCAAGGATGACACGCACATCCGTG
Zv7_scaffold2561        AGAAGATTAGCATGGCCCCTGCGCAAAGGAACCTGATGGATCTTCT//CGAAGATTCTCCCGCAAGAGGCGAAAGGATGACACGCAAATCCGTG
chr4                    AGAAGATTAGCATGGCCCCTGCGCAAAGGAACCTGATGGATCTTCT//CGAAGATTCTCCCGCAAGAGGCGCAAGGATGACACGCAAAAGAATG
Zv7_scaffold2559        AGAAGATTAGCATGGCCCCTGCGAAAAGGAACCTGATGGATCTTCT//CGAAGATTCTCCCGCAAGAGGCGCAAGGATGACACGCAAATCCGTG
Zv7_scaffold2561        AGAAGATTAGCATGGCCCCTGCGAAAAGGAACCTGATGGATCTTCT//CGAAGATTCTCCCGCAAGAGGCGAAAGGATGACACGCAAATCCGTG
chr3                    AGAAGATTAGCATGGCCCCTGCGAAAAGGAACCTGATGGATCTTCT//CGAAGATTCTCCCGCAAGAGGCGCAAGGATGACACGCAAATCCGTG
Zv7_scaffold2490        AGAAGATTAGCATGGCCCCTGCGCAAAGGAACCTGATGGATCTTCT//CGAAGATTCTCCCGCAAGAGGCGCAAGGATGACACGCAAATCCGTG
chr3                    AGAAGATTAGCATGGCCCCTGCGAAAAGGAACCTGATGGATCTTCT//CGAAGATTCTCCCGCAAGAGGCGCAAGGATGACACGCAAATCCGTG
chr3                    AGAAGATTAGCATGGCCCCTGCGAAAAGGAACCTGATGGATCTTCT//CGAAGATTCTCCCGCAAGAGGCGCAAGGATGACACGCAAATCCGTG
chr3                    AGAAGATTAGCATGGCCCCTGCGAAAAGGAACCTGATGGATCTTCT//CGAAGATTCTCCCGCAAGAGGCGCAAGGATGACACGCAAATCCGTG
chr3                    AGAAGATTAGCATGGCCCCTGCGAAAAGGAACCTGATGGATCTTCT//CGAAGATTCTCCCGCAAGAGGCGCAAGGATGACACGCAAATCCGTG
Zv7_scaffold2561        AGAAGATTAGCATGGCCCCTGCGAAAAGGAACCTGATGGATCTTCT//CGAAGATTCTCCCGCAAGAGGCGAAAGGATGACACGCAAATCCGTG
Zv7_scaffold2602        AGAAGATTAGCATGGCCCCTGCGAAAAGGAACCTGATGGATCTTCT//CGAAGATTCTCCCGCAAGAGGCGCAAGGATGACACGCAAATCCGTG
Zv7_NA108               AGAAGATTAGCATGGCCCCTGCGAAAAGGAACCTGATGAATCTTCT//CGAAGATTCTCCCGCAAGAGGCGAAAGGATGACACGCAAATCCGTG
chr3                    AGAAGATTAGCATGGCCCCTGCGAAAAGGAACCTGATGGATCTTCT//CGAAGATTCTCCCGCAAGAGGCGCAAGGATGACACGCAAATCCGTG
chr14                   AGAAGATTAGCATGGCCCCTGCGAAAAGGAACCTGATGGATCTTCT//CGAAGATTCTCCCGCAAGAGGCGAAAGGATGACACGCAAATCCGTG
chr4                    AGAATATTAGCATGGCCCCTGCGAAAAGGAACCTGATGGATCTTCT//CGAAGATTCTCCCGCAAGAGGCGAAAGGATGACACGCAAATCCGTG
Zv7_scaffold2561        AGAAGATTAGCATGGCCCCTGCGAAAAGGAACCTGATGGATCTTCT//CGAAGATTCTCCCGCAAGAGGCGCAAGGATGACACGCAAATCCGTG
chr12                   AGAAGATTAGCATGGCCCCTGCGAAAAGCAACCTGATGGATCTTCT//CGAAGATTCTCCCGCAAGAGGCGCAAGGATGACACGCAAATCCGTG
Zv7_scaffold2558        AGAAGATTAGCATGGCTCCTGCGAAAAGGAACCTGATGGATCTTCT//CGAAGATTCTCCCGCAAGAGGCGCAAGGATGACACGCAAATCCGTG
chr5                    AGAAGATTAGCATGGCCCCTGCGAAAAGGAACCTGATGGATCTTCT//CGAAGATTCTCCCGCAAGAGGCGCAAGGATGACACGCAAATACGTG
chr4                    AGAAGATTAGCATGGCCCCTGTGAAAAGGAACCTGATGGATCTTCT//CGAAGATTCTCCCGCAAGAGGCGAAAGGATGACACGCAAATCTGTG
chr16                   AGAAGATTAGCATGGCCCCTACGCGAAGGAACCTGATGGATCTTCT//CGAAGATTCTCCCGCAAGAGGCGCAAGGATGACACGCAAATCCGTG
chr4                    AGCAGATTGAGGAGGAGACAGAATGGAGGAACCTGATGGATCTTCT//CGAAGATTCTCCCGCAAGAGGCGAAAGGATGACATGAAAATCCGTG
Zv7_scaffold2561        AGCAGATTGAGGAGGAGACAGAATGGAGGAACCTGATGGATCTTCT//CGAAGATTCTCCCGCAAGAGGCGAAAGGATGACACGCAAATCCGTG
Zv7_scaffold2497        AGCAGATTGAGGAGGAGACAGAATGGAGGAACCTGATGGATCTTCT//CGAAGATTCTCTCGCAGGGAGCGCAAGGATGACACGCAAATCCGTG
```

Dada-U6_DPu(Daphnia pulex)

```
U6 snRNA                AGAAGATTAGCATGGCCCCT------------------------------------------------GCGCAA-GGATGACACGCAAAATCGTG
ACJG01005051            AGAAGACTAGCATGGCCCCTGCGCAAAGGCTGGGGCGTAATCTTCT//
ACJG01014537            AGAAGACTAGCATGGCCCCTGCGCAAAGGCTGGGGCGTAATCTTCT//
ACJG01016944            AGAAGACTAGCATGGCCCCTGCGCAAAGGCTGGGGCGTAATCTTCT//
ACJG01014537            AGAAGACTAGCATGGCCCCTGCGCAAAGGCTGGGGCGTAATCTTCT//
ACJG01003668            AGAAGAAACGGCGCCGCGGAAGGCTGAGGCTGGGGCGTAATTTTCT//
ACJG01009737            AGAAGAAACGGCGCCGCGGAAGGCTGAGGCTGGGGCGTAATTTTCT//
ACJG01005050                                                          //AGAAGATTGGGGGACAAGCAGCGCAAAGGATGACACGCAAAATCGTT
ACJG01005766                                                          //AGAAGATTGGGGGACAAGCAGCGCAAAGGATGACACGCAAAATCGTT
ACJG01005767                                                          //AGAAGATTGGGGGACAAGCAGCGCAAAGGATGACACGCAAAATCGTT
ACJG01011249                                                          //AGAAGATTGGGGGACAAGCAGCGCAA-CGATGACACGCAAA-TCGTG
```

Dada-U6_CT(Capitella teleta)

```
U6 snRNA                AGAAGATTAGCATGGCCCCT------------------------------------------------GCGCAAGGATGACACGCAAATTCGTG
scaffold_739            AGAAGATTAGCATGGCCCCTGCGCAAAGGAACCCGGCCAATCTTCT//AAACTAGAGTTGTGCGCAAGGCGCAAGGATGACACGCAAATTCGTG
scaffold_1222           AGAAGATTAGCATGGCCCCTGCGCAAAGGAACCCGGCCAATCTTCT//
scaffold_7139           AGAAGATTAGCATGGCCCCTGCGCAAAGGAACCCGGCCAATCTTCT//
scaffold_8138           AGAAGATTAGCATGGCCCCTGCGCAAAGGAACCCGGCCAATCTTCT//
scaffold_99             AGAAGATTAGCATGGCCCCTGCGCAAAGGAACCCGGCCAATCTTCT//
scaffold_114            AACATAATGAGCCAGATGAGTTTCCAAGGAACCCGGCCAATCTTCT//
scaffold_11596          CTGCCATAGAATTGGAACTTCTGTTAAGGAACCCGGCCAATCTTCT//
scaffold_1119                                                         //AAACTAGAGTTGTGCGCAAGGCGCAAGGATGACACGCAAATTCGTG
scaffold_133                                                          //AAACTAGAGTTGTGCGCAAGGCGCAAGGATGACACGCAAATTCGTG
scaffold_5                                                            //AAACTAGAGTTGTGCGCAAGGCGCAAGGATGACACGCAAATTCGTG
scaffold_11596                                                        //AAACTAGAGTTGT-------GCGCAA----GACACGCAAATTCGTG
```
