# Supplementary material for: A Superfamily of DNA Transposons Targeting Multicopy Small RNA Genes
Source: PLoS One. 2013 Jul 9;8(7):e68260. doi: 10.1371/journal.pone.0068260 (PMC3706591; doi:10.1371/journal.pone.0068260)
Supplement: Figure S3 — Tandem insertions of Dada-U1A_DR and Dada-U1B_DR transposons. The sequences of Dada-U1A_DR are colored in blue, of Dada-U1B_DR in magenta, and of TSD in red. (PDF) [file pone.0068260.s003.pdf]

Figure S3.

```
U1 snRNA gene     CACTCCGGCCACGCTGACCCCTGCGAATTCCCCAAATGTGGGAATCTC
Dada-U1B_DR       CACGACTGTAGAAAGAACCTCT 3’
chr12             CACGACTGTAGAAA--------GCGAATGGTGATCGACCTGTACGACG
Dada-U1A_DR                                5’ GGCGATCGACCTGTACGGCG
Dada-U1A_DR       CACGTTCAGTGGGCAGATGTCT 3’
chr3              CACGTTCCCTGGGCAGATGTCC------GGCGATCGACCTGTACGGCG
```
