# Supplementary material for: A Superfamily of DNA Transposons Targeting Multicopy Small RNA Genes
Source: PLoS One. 2013 Jul 9;8(7):e68260. doi: 10.1371/journal.pone.0068260 (PMC3706591; doi:10.1371/journal.pone.0068260)
Supplement: Figure S4 — Insertions of Dada-tA_DR and Dada-tA_OL. TSD are colored in red and Dada transposons are in blue. Anticodons in the tRNA genes are underlined. (PDF) [file pone.0068260.s004.pdf]

# Figure S4.

```
Dada-tA_DR(Danio rerio)
tRNA-Ala-GCT    GGGGAATTAGCTCAAATGGTAGAGCGCTCGCTTAGCAT----------------------------GCGAGAGGTAGCGGGATCGATGCCCGCATTCTCCA
Chr25           GGGGAATTAGCTCAAATGGTAGAGCGCTCGCTGCGCAAAGGAAGGGGC//GATGCTGCAGGCGCATGCGAGAGGTAGCGGGATCGATGCCCGCATTCTCCA
Chr25           GGGGAATTAGCTCAAATGGTAGAGCGCTCGCTGCGCAAAGGAAGGGGC//GATGCTGCAGGCGCAAGCGAGAGGTAGCGGGATCGATGCCCGCATTCTCCA
Chr25           GGGGAATTAGCTCAAATGGTAGAGCGCTCGCTGCGCAAAGGAAGGGGC//GATGCTGCAGGCGCAAGCGAGAGGTAGCGGGATCGATGCCCGCATTCTCCA
Chr25           GGGGAATTAGCTCAAATGGTAGAGCGCTCGCTTAGCATAGGAAGGGGC//GATGCTGCAGGCGCAAGCGAGAGGTAGCGGGATCGATGCCCGCATTCTCCA
Chr3            GGGGAATTAGCTCAAATGGTAGAGCGCTCGCTGCGCAAAGGAAGGGGC//GATGCTGCAGGCGCAGGCGAGAGGTGGCGGGATCGATACACGCATTCTCCA
Chr3            GGGGAATTAGCTCAAATGGTAGAGCTCTCGCTGCGCAAAGGAAGGGGC//GATGCTGCAGGCGCAAGCGAGAGGTAGCGGGATCGATGCCCGCATTCTCCA

Dada-tA_OL(Oryzias latipes)
tRNA-Ala-GCT    GGGGAATTAGCTCAAATGGTAGAGCGCTCGCTTAGCAT----------------------------GCGAGAGGTAGCGGGATCGATGCCCGCATTCTCCA
BAAF04121516    GGGGAATTAGCTCAAATGGTAGAGCACTCGCTGCGCAAAGGCGGGGGG//
BAAF04121512    GGGGAATTAGCTCAAATGGTAGAGCACTTGCTGCGCAAAGGCGGGGGG//
BAAF04121513    GGGGAATTAGCTCAAATGGTAGAGCACTCGCTGAGCAAAGGCGGGGGG//
BAAF04097840    GGGGAATTAGCTCAAATGGTAGAGCGCTCGCTTTGCATAAGCAGGGGG//
chr20                                                           //ACGCCAGTAGGCGCAAGTGAGAGGTAGGGGGGTCGATCCCCTCATTCTCCA
```
